# Detection of vertical root fractures in endodontically treated teeth in the absence and in the presence of metal post by cone-beam computed tomography

**DOI:** 10.1186/s12903-016-0207-y

**Published:** 2016-04-14

**Authors:** Rebeca Ferraz de Menezes, Natália Costa de Araújo, Joedy Maria Costa Santa Rosa, Vanda Sanderana Macêdo Carneiro, Alexandrino Pereira dos Santos Neto, Vânio Costa, Lara Marques Moreno, Jéssica Meirinhos Miranda, Diana Santana de Albuquerque, Mônica Albuquerque, Roberto Alves dos Santos, Marleny Elizabeth Márquez de Martínez Gerbi

**Affiliations:** Department of Endodontics, Faculty of Dentistry, University of Pernambuco, 1650 Newton Cavalcanti Avenue, Zipe Code: 54753-020 Camaragibe, PE Brazil; Department of Dentomaxillofacial Radiology, Faculty of Dentistry, Federal University of Alagoas, Lourival Melo Mota Avenue, S/N - Tabuleiro dos Martins, Zipe Code: 57072-900 Maceió, AL Brazil; Department of Restorative Dentistry, Faculty of Dentistry, University of Pernambuco, 1650 Newton Cavalcanti Avenue, Zipe Code: 54753-020 Camaragibe, PE Brazil

**Keywords:** Vertical root fracture, Cone-Beam Computed Tomography, Dental posts

## Abstract

**Background:**

Aim of this study was to investigate the influence of gutta-percha and metallic posts on the efficiency of Cone Beam Computed Tomography (CBCT) in diagnosing Vertical Root Fracture (VRF).

**Methods:**

Forty-eight teeth were divided into 3 experimental and 3 control groups. The teeth of the first experimental group and the first control group received neither gutta-percha nor metal posts. The teeth of the second experimental group and the second control group were filled with gutta-percha, and the teeth of the third experimental group and the third control group were filled with the metal posts. The teeth of the experimental groups were artificially fractured. The teeth were evaluated through images taken by a Prexion scanner with a 0.1 mm resolution. Fisher’s exact test was used to measure the following values: sensitivity, false negative, specificity, false positive and accuracy for the VRF detection through the scanner. Three observers calibrated and blinded to the protocol evaluated the images.

**Results:**

The inter-observer Kappa coefficient was 0.83. The presence of posts and gutta-percha reduced the sensitivity and the accuracy in detecting the VRF. Regarding to the sensitivity (*p* = 0.837, *p* = 0.304, *p* = 0.837 for evaluator 1, 2 and 3, respectively) and specificity (*p* = 0.162, *p* = 0.056, *p* = 0.062 for evaluator 1, 2 and 3, respectively), Fisher’s exact test showed no statistically significant difference among the evaluated groups. However, a significant difference was observed in relation to the accuracy in the results of evaluator 2 (*p* = 0.03), which showed a much lower accuracy for the post group (50 %) than for the Nonfilled group (93.8 %).

**Conclusions:**

The Prexion tomograph was precise in detecting vertical root fractures and the CBCT diagnostic ability was not influenced by the presence of posts or gutta-percha.

## Background

Vertical root fracture (VRF) may be defined as a complete or incomplete fracture that starts from the root at any level and is usually directed buccolingually. The fracture is located in the root portion of the tooth and may extend coronally towards the cervical periodontal attachment [1].

When a VRF occurs, it extends to the periodontal ligament. The fracture can get in contact with the oral cavity through gingival sulcus therefore foreign matter, food debris and bacteria can have access to the fractured area. An inflammatory process is induced, resulting in a breakdown of the periodontal ligament, loss of alveolar bone, and granular tissue formation [2].

The signs, symptoms and/or radiographic features of VRF are not always accurate, and VRF can often be confused with a failure in endodontic treatment and even periodontal disease; however, when VRF occurs, the most common signs and symptoms in endodontically treated teeth are pain, swelling, sinus tract and an isolated periodontal pocket that is deep and narrow. The radiographic features are represented by the widening of the periodontal ligament, vertical bone loss, and periradicular bone loss (bone loss halo) [3, 4]. Although the diagnosis of a root fracture is a challenge for an endodontist, the correct diagnosis of the VRF is important for assessing the prognosis and determining the appropriate treatment for the tooth [5].

Imaging exams are very important tools in the diagnosis of VRF. The periapical radiograph is widely used as the first test to help diagnose these fractures; however, due to its limitations, such as the inability to provide a two-dimensional image with overlapping structures adjacent to the tooth and the need of the central X-ray beam to be located parallel to the fracture line, X-rays must be taken at different angles to complete the diagnosis [6–10]. To minimize these limitations and provide radiographic images more accurately, cone beam computed tomography (CBCT) scans, an imaging technique capable of providing three dimensional (3D) images, are being increasingly used for dental diagnoses [11, 12]. The superiority of CBCT images when compared to other radiographic methods in diagnosing VRFs has been well reported [7, 13–17].

The detection of root fractures by CBCT has already been demonstrated by previous studies [4, 5, 11, 12, 18–23]. As VRF has been considered as one of the major reasons for the extraction of root filled teeth, it is important to assess the influence of the gutta-percha root fillings on the diagnosis of VRF [24]. Besides, VRF can be found in endodontically treated tooth with a metal post and in cases in which there are metallic objects associated with the involved teeth, artefacts can appear on tomographic images, rendering the interpretation of the exam difficult in the diagnosis of root fracture. However, a limited number of studies assessing the influence of imaging artefacts on the diagnosis of root fractures can be found in the literature [19–21].

Furthermore, few studies worked with Prexion tomograph (TeraRecon, Tokyo, Japan) with a resolution voxel of 0.1 mm, and no studies have assessed the influence of the presence of a metal post on detection of VRFs. Based on this, the present study investigated the efficiency of Prexion tomograph in diagnosing VRFs in endodontically treated teeth, with and without metal post.

## Methods

This study was conducted with the approval of the Ethics Committee of the State University of Pernambuco (Protocol #0251.0.097.000-11).

Forty-eight single-rooted human teeth, which were extracted for therapeutic reasons, were inspected by transillumination with the help of a magnifying glass (magnifying lens 4x) to confirm the absence of a radicular fracture. Periapical radiographs were taken to exclude teeth with calcification and internal root resorption. Teeth with incomplete root formation were also excluded from the study.

An access opening was made, and the root canals were prepared with the ProTaper rotary system (Dentsply Maillefer, Tulsa, OK) up to size F3 by a single endodontist.

**Fig. 1:**
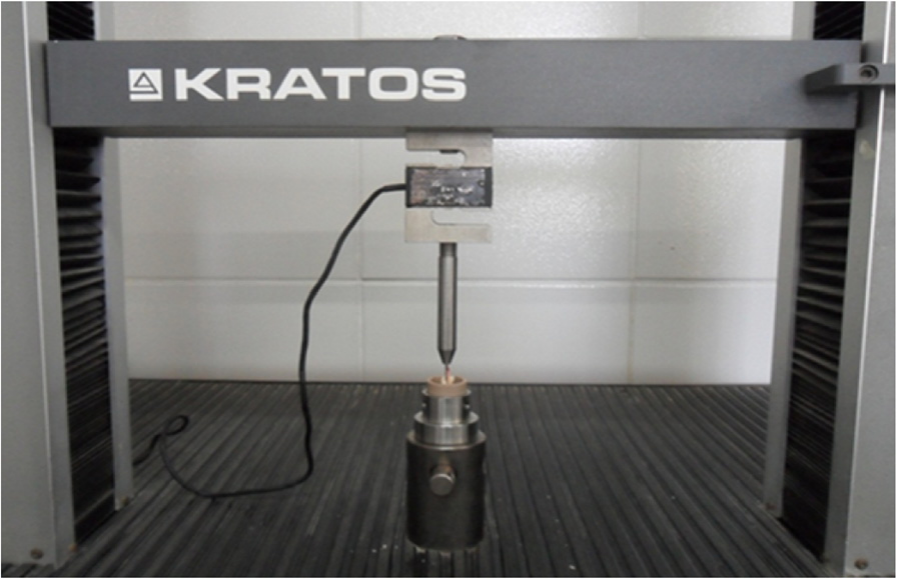
Machine used to induce vertical root fractures

Forty-eight teeth were randomly coded and divided into 3 control and 3 experimental groups. Table 1 shows the name of the groups, the name of the intracanal materials and sample size of each group. Periapical radiographs were obtained to verify the adaptation of gutta-percha cone F3 and the metal post in the root canal after the preparation. After this step, the cones and the posts were removed and all of the teeth of the experimental groups (24 teeth) were fractured. Wax and acrylic resin dyes were made for adapting and fixing the teeth to receive the fractures [18]. The VRFs were made according to the methodology of Neves et al. [19]. The technique to promote the fracture of teeth consisted of applying a vertically directed force using a tapered tip tool tailored to the Clinical Trials machine (Kratos Equipamentos, São Paulo, Brazil) directly over the entries of the root canals (Fig. 1). The fractured specimens were reinspected by transillumination with the help of a magnifying glass (magnification 4x) to confirm the presence of a VRF. The gutta-percha and the metal posts were reinserted in their respective groups. Because cement might flow to the fracture line, the cementation procedure was avoided.

**Table 1:** The name of the groups, the name of the intracanal materials and sample size of each group

Name of the groups	Name of the Intracanal materials	Sample size (n)	Sample size (n)
		Control Groups (Without VRF)	Experimental Groups (With VRF)
Nonfilled Group	Neither gutta-percha nor a metal post	8	8
Gutta-percha Group	Gutta-percha	8	8
Post Group	Metal post	8	8

### CBCT scans

For the acquisition of CBCT images, forty-eight teeth were divided randomly into groups of four. Each tooth was coated with a layer of wax and placed in an empty socket of a dry human mandible. The mandible was coated with three layers of dental wax buccally and lingually to simulate soft tissue and to reduce artefacts in the images [20].

The mandible was placed in a plastic cylinder box containing water to simulate a clinical situation [21, 22].

**Fig. 2:**
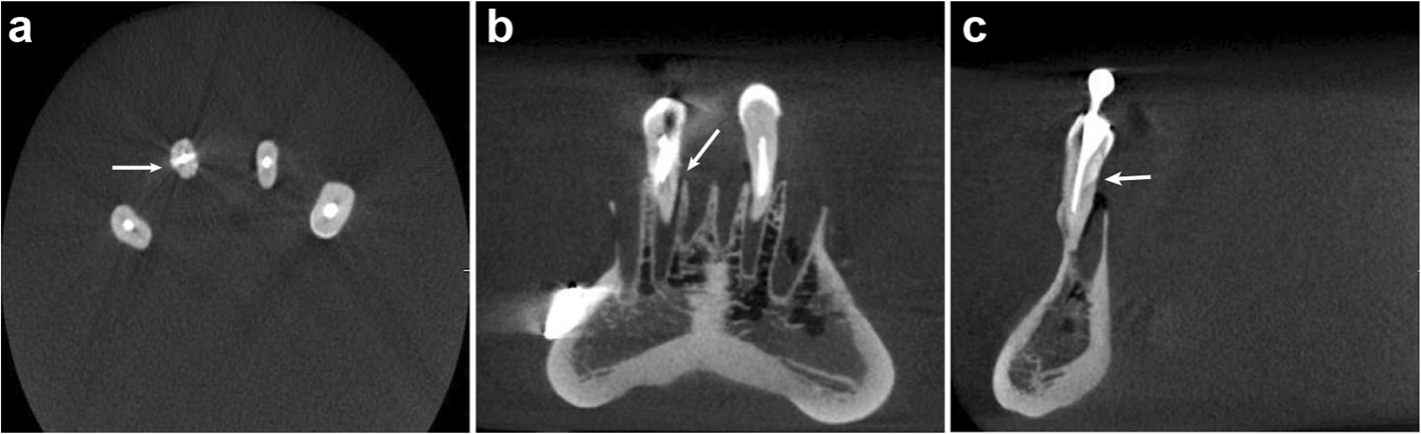
Prexion CBCT reconstructions. The vertical root fracture is observed in Axial (**a**), Coronal (**b**), and Sagital (**c**) slices in a tooth with a metal post

The sample was scanned by a Prexion tomograph (90 kV, 4 mA, 5-cm field of view, 37 s for acquisition). Prexviewer software was used for image analysis. The data set was exported in DICOM format and the voxel size was 0.1 mm resolution. The data were reconstructed with cuts in the axial, coronal and sagittal planes (Fig. 2), and the images were analysed by three calibrated radiologists, blinded to the protocol, with 10 years of experience in CBCT. The calibration consisted of the identification of the existence of fractures in 45 tomographic images of root-filled (gutta-percha and metal post) and nonfilled teeth that did not belong to the study sample. The same observation was repeated after 20-day interval. All of the images were analysed on a 27-in. LED screen computer in a darkened room. Each observer was asked about the presence or absence of a fracture using a dichotomous scale (1- tooth fractured / 0- tooth not fractured).

### Statistical analysis

The data were analysed using SPSS version 21. Fisher’s exact test was used to measure the following values: sensitivity, false negative, specificity, false positive and accuracy for the VRF detection through the Prexion scanner. To evaluate the inter-observer agreement level, the kappa coefficient was obtained. The margin of error used in the statistical tests was 5 %, and the intervals were obtained with 95.0 % reliability.

## Results

The inter-observer kappa coefficient was 0.83, indicating very good inter-observer agreement. Table 2 shows the results of the following values: sensitivity, specificity, accuracy, false positive and false negative of evaluators 1, 2 and 3 for the presence or absence of fractures.

**Table 2:** Sensitivity, specificity, accuracy, false positives, and false negatives by evaluator 1, 2 and 3 in relation to VRF occurrence

Evaluator	Group	Sensitivity		Specificity		Accuracy		False positive		False negative	
		n	%	n	%	n	%	n	%	n	%
1	• Nonfilled	7	87.5	6	75.0	13	81.2	2	25	1	12.5
	• Gutta-percha	5	62.5	7	87.5	12	75.0	1	12.5	3	37.5
	• Post	6	75.0	3	37.5	9	56.3	5	62.5	2	25
	P value	*p* = 0.837		*p* = 0.162		*p* = 0.375					
2	• Nonfilled	8	100	7	87.5	15	93.8	1	12.5	- -	
	• Gutta-percha	5	62.5	6	75	11	68.8	2	25	3	37.5
	• Post	6	75.0	2	25	8	50	6	75	2	25
	P value	*p* = 0.304		*p* = 0.056		*p* = 0.030					
3	• Nonfilled	7	87.5	7	87.5	14	87.5	1	12.5	1	12.5
	• Gutta-percha	6	75	7	87.5	13	81.3	1	12.5	2	25
	• Post	5	62.5	3	37.5	8	50	5	62.5	3	37.5
	P value	*p* = 0.837		*p* = 0.062		*p* = 0.075					

The results show that the sensitivity was higher in the Nonfilled group and that the Post group always had the highest percentage of false-positives, but these did not reach statistical significance. The only significant difference was observed in relation to the accuracy in the results of evaluator 2 (*p* = 0.03), which showed a much lower accuracy for the Post group (50 %) than for the Nonfilled group (93.8 %).

## Discussion

This study investigated the ability of the CBCT scans in detecting VRF in endodontically treated teeth in the absence and presence of metal post. The results (Table 2) showed an overall higher accuracy and sensitivity in Nonfilled group. On the other hand, the Post group had the highest percentage of false positives because the presence of metal caused a greater loss of data in the image reconstruction process and resulted with the formation of beam hardening artefacts.

Based on previous studies [19, 25], the present study used teeth which were filled with gutta-percha cones as filling material without any root canal sealer. No root sealer was used with the intracanal materials before the induction of the fracture because it would invalidate the use of the same teeth under different conditions. Moreover, root sealer was not used after the induction of the fracture because this could displace the root fragments and even flow to the fracture line, indicating its presence.

Periapical radiographs are not reliable methods for diagnosing VRFs [4, 7, 23]. Reliability is reduced further when the VRFs are present in teeth with gutta-percha or a metal post. Radiopaque material might appear as dark areas or stripes around the endodontic materials that could simulate lines of fractures in images of healthy teeth [10].

In the present study, periapical radiography was not included because the main objective here was to evaluate the influence of different intracanal materials in the detection of VRFs using Prexion Tomograph imaging. The results of current study (Table 2) indicate that Prexion tomograph with a 0.1 mm voxel resolution is effective for the diagnosis of VRFs. These results were consistent with the results observed previously [7, 15, 16, 25–28]. Yet, many of these studies did not associate the influence of the presence of gutta-percha and metal posts with the ability of CBCT to diagnose VRFs.

Other studies have shown that tomographs with smaller voxel sizes are more effective and more accurate to detect VRFs [9, 10, 15, 25–27]. In the present study, an important aspect was the choice of the scanner. The Prexion tomograph has a small voxel size, 0.1-mm voxel, and this makes it more accurate to diagnose VRFs. It has also been suggested that the root canal contents (presence of a root filling, metal post) should guide the choice of voxel size [29].

Hassan et al. [7] compared the accuracy of CBCT and periapical radiographs to detect the presence of VRFs in endodontically treated and untreated teeth. They concluded that the presence of gutta-percha significantly reduced the specificity (*p* = 0.016); however, the overall accuracy of the tomograph to diagnose VRF was not influenced by the presence of gutta-percha.

Clinical researches have concluded that the presence of gutta-percha in the root canal did not significantly influence the sensitivity, specificity or accuracy in the diagnosis of vertical root fractures. Therefore, even before the presence of filling material, CBCT was able to accurately diagnose VRFs [15, 26]. These researches corroborate with the results of present study that showed the presence of gutta-percha in teeth negatively influenced the diagnosis of VRFs when compared with nonfilled group; however, no significant differences were found.

Additionally, previous study compared the diagnostic accuracy of CBCT with periapical radiography in detecting artificially prepared VRFs in the presence of a gutta-percha root filling in human teeth. This study indicated that periapical radiographs and CBCT were not accurate in detecting the presence or absence of simulated VRF. The imaging artefacts caused by the gutta-percha root filling within the root canal most likely resulted in an overestimation of VRF with CBCT and the overall inaccuracy of this system. Despite the three-dimensional nature of the reconstructed CBCT images, the poor resolution of CBCT and artefacts caused by gutta-percha contributed to the inaccuracy of CBCT [30].

Melo et al. [25] evaluated the influence of the presence of metal posts and gutta-percha on the ability of CBCT with 2 voxel resolutions (0.3 mm and 0.2 mm) to diagnose longitudinal root fractures. They concluded that the presence of gutta-percha and metal posts reduced the sensitivity and specificity at both voxel resolutions. The value of sensitivity for the scanner with the 0.2 mm voxel resolution was significantly higher for the Nonfilled group (*p* < .05) than for the Gutta-percha and Post groups. The results of this study corroborated these findings. Table 2 shows that the overall accuracy for detecting the presence of VRFs in the Gutta-percha group and the Post group was always less than the accuracy in the Nonfilled group. Moreover, the results of evaluator 2 showed that there was a significant difference regarding the accuracy among the three groups (*p* = 0.030), which ranged from 93.8 % in the Nonfilled group to 50 % for the Post group. For the Post group, the false-positive rates were always higher than those of the other two groups, at 62.5 %, 75 % and 62.5 %, according to evaluators 1, 2 and 3, respectively.

The present study showed that the presence of a metal post in the root canal increases the probability of artefacts in the images of CBCT scans, as it was revealed in the work of Bechara et al. [31]. These artefacts could account for the cases of false-positive and false-negative results. In this study, the false-positive values were higher in the Post group according to all three observers.

Other studies also revealed that the presence of a metal post in endodontically treated teeth negatively influenced the diagnosis of VRFs due to the presence of artefacts in the fracture lines [14, 32, 33].

As the prognosis of teeth with VRFs is frequently poor, tooth extraction is usually the treatment required [3, 6]. A detailed diagnosis of these fractures is very important and ideally should be performed with a scanner of a small voxel size.

Although CBCT images are quite effective, the present study showed that in specific cases (in the presence of metal post, for example), even with CBCT, diagnosing root fractures is not easy. Besides, clinical signs (teeth with mobility, sinus tract and an isolated periodontal pocket) and symptoms (pain while chewing) are important in the diagnosis of the VRFs [6, 9, 34]. However, such conditions are not amenable to simulation, constituting a limitation of in vitro studies.

In the present study, only the Prexion tomograph was tested and only-single rooted teeth were used. Thus, in vitro studies with multiple-rooted teeth, other tomography scans and clinical researches should be developed.

## Conclusion

The presence of gutta-percha and metal posts in root canals reduced the sensitivity and accuracy of the tomograph in detecting VRFs. The sensitivity was higher in the nonfilled group and the post group had the highest percentage of false-positives results, but these results did not reach statistical significance. Thus, Prexion tomograph with 0.1 mm resolution is a precise, accurate and useful method to detect VRFs in the absence and presence of metal post.

## Ethics approval and consent to participate

This study was conducted with the approval of the Ethics Committee of the State University of Pernambuco (Protocol #0251.0.097.000-11).

The consent was not required as the teeth were removed as part of routine therapy.

## Availability of data and materials

All the data supporting our findings is contained within the manuscript.

## Abbreviations

CBCT: Cone Beam Computed Tomography
VRF: Vertical Root Fracture
3D: Three Dimensional
